# Neutralizing Monoclonal Antibodies That Target the Spike Receptor Binding Domain Confer Fc Receptor-Independent Protection against SARS-CoV-2 Infection in Syrian Hamsters

**DOI:** 10.1128/mBio.02395-21

**Published:** 2021-09-14

**Authors:** Wen Su, Sin Fun Sia, Aaron J. Schmitz, Traci L. Bricker, Tyler N. Starr, Allison J. Greaney, Jackson S. Turner, Bassem M. Mohammed, Zhuoming Liu, Ka Tim Choy, Tamarand L. Darling, Astha Joshi, Ka Man Cheng, Alvina Y. L. Wong, Houda H. Harastani, John M. Nicholls, Sean P. J. Whelan, Jesse D. Bloom, Hui-Ling Yen, Ali H. Ellebedy, Adrianus C. M. Boon

**Affiliations:** a School of Public Health, Li Ka Shing Faculty of Medicine, The University of Hong Kong, Hong Kong SAR, China; b Department of Pathology and Immunology, Washington University School of Medicine in St. Louis, St. Louis, Missouri, USA; c Department of Internal Medicine, Washington University School of Medicine in St. Louis, St. Louis, Missouri, USA; d Department of Microbiology, Washington University School of Medicine in St. Louis, St. Louis, Missouri, USA; e Fred Hutchinson Cancer Research Center, Seattle, Washington, USA; f Department of Pathology, LKS Faculty of Medicine, The University of Hong Kong, Hong Kong, Hong Kong; g Howard Hughes Medical Institute, Seattle, Washington, USA; Harvard Medical School

**Keywords:** COVID-19, SARS-CoV-2, Syrian hamster, transmission, monoclonal antibodies

## Abstract

The severe acute respiratory syndrome coronavirus 2 (SARS-CoV-2) spike protein is the main target for neutralizing antibodies. These antibodies can be elicited through immunization or passively transferred as therapeutics in the form of convalescent-phase sera or monoclonal antibodies (MAbs). Potently neutralizing antibodies are expected to confer protection; however, it is unclear whether weakly neutralizing antibodies contribute to protection. Also, their mechanism of action *in vivo* is incompletely understood. Here, we demonstrate that 2B04, an antibody with an ultrapotent neutralizing activity (50% inhibitory concentration [IC_50_] of 0.04 μg/ml), protects hamsters against SARS-CoV-2 in a prophylactic and therapeutic infection model. Protection is associated with reduced weight loss and viral loads in nasal turbinates and lungs after challenge. MAb 2B04 also blocked aerosol transmission of the virus to naive contacts. We next examined three additional MAbs (2C02, 2C03, and 2E06), recognizing distinct epitopes within the receptor binding domain of spike protein that possess either minimal (2C02 and 2E06, IC_50_ > 20 μg/ml) or weak (2C03, IC_50_ of 5 μg/ml) virus neutralization capacity *in vitro*. Only 2C03 protected Syrian hamsters from weight loss and reduced lung viral load after SARS-CoV-2 infection. Finally, we demonstrated that Fc-Fc receptor interactions were not required for protection when 2B04 and 2C03 were administered prophylactically. These findings inform the mechanism of protection and support the rational development of antibody-mediated protection against SARS-CoV-2 infections.

## INTRODUCTION

The ongoing coronavirus disease (coronavirus disease 2019 [COVID-19]) pandemic, caused by the novel coronavirus severe acute respiratory syndrome coronavirus 2 (SARS-CoV-2), has resulted in the loss of millions of lives and trillions of dollars within 1 year from its emergence. Safe and effective vaccines are considered the ultimate remedy for the global social and economic disruption caused by the pandemic. Vaccines aim to elicit neutralizing antibodies that target the spike (S) protein of SARS-CoV-2, which mediates viral attachment and membrane fusion of the virus. However, a thorough understanding of the immune correlates of protection against this novel coronavirus remains lacking. Addressing this knowledge gap is urgently needed to complement the ongoing vaccine development and evaluation efforts.

Neutralizing antibodies targeting the receptor binding domain (RBD) or N-terminal domain (NTD) of the S protein may confer protection against SARS-CoV-2 by blocking viral infection of cells by a variety of mechanisms, including preventing viral attachment to cellular angiotensin-converting enzyme 2 (ACE2) receptors and other mechanisms (1–8). The RBD is immunodominant and is usually the main target of the neutralizing activity in the immune sera of COVID-19 patients (9). Both neutralizing and nonneutralizing antibodies may mediate protection via Fc-mediated effector functions by interacting with Fcγ receptors (FcγR) on NK cells and phagocytes to trigger antibody-dependent cellular cytotoxicity (ADCC) and antibody-dependent cellular phagocytosis (ADCP), respectively. Studies of influenza viruses showed that strain-specific neutralizing monoclonal antibodies (MAbs), targeting head domain of the hemagglutinin (HA), conferred *in vivo* protection via a Fc-independent mechanism. In contrast, optimal *in vivo* protection mediated by broadly cross-reactive MAbs, targeting the stalk domain of the HA, or nonneutralizing MAbs, required Fc-mediated effector functions (10, 11). Antibodies may also interact with the complement system and activate the complement-dependent cytotoxicity (CDC). The potential for antibody-dependent enhancement of infection or disease (ADE) in coronavirus immunopathogenesis has been suggested due to the observed association of time-dependent progression into severe disease and the kinetics of neutralizing antibody development (12, 13). However, no ADE has been observed in animals or humans after receiving vaccines against SARS-CoV-2 or from recovered COVID-19 patients thus far.

Neutralizing monoclonal antibodies derived from convalescent COVID-19 patients have demonstrated potent prophylactic or therapeutic effect in nonhuman primates, hamsters, and mice (4, 6, 14–16). Several of these MAbs are being evaluated as potential therapeutics in human studies (17). Vaccination or natural infection will induce a spectrum of antibodies that vary in their ability to neutralize SARS-CoV-2. How such differences influence the *in vivo* protective capacity of such antibodies remains unknown. Further, the significance of Fc-mediated effector functions has not been fully investigated. We previously generated a panel of RBD-targeting murine MAbs with different binding affinities and neutralizing activities and demonstrated the protective effect of a highly potent neutralizing MAb in a murine model of SARS-CoV-2 (18). To delineate the roles of neutralizing and nonneutralizing antibodies against SARS-CoV-2, we compared the protective effect of a potently neutralizing MAb (2B04) (50% inhibitory concentration [IC_50_] of 0.04 μg/ml), a weakly neutralizing MAb (2C03) (IC_50_ of 5 μg/ml), and two minimal neutralizing MAbs (2C02 and 2E06) (IC_50_ > 20 μg/ml) in Syrian hamsters and determined the role of Fc receptor interactions on protection. We find that the potently neutralizing MAb 2B04 reduces viral burden and disease severity when given as a pre- or postexposure therapy. Preexposure administration of the weakly neutralizing MAb 2C03 also reduced viral load and weight loss after challenge, while the minimal neutralizing MAbs 2C02 and 2E06 did not confer protection. The protective effect of 2B04 and 2C03 was not dependent on Fc receptor engagement. Finally, we show that prophylactic treatment of 2B04 reduced viral load in the nasal turbinates of inoculated donors and prevented onward transmission of SARS-CoV-2 to naive hamsters by air. Overall, these results suggest that MAbs targeting RBD of S may confer protection against SARS-CoV-2 *in vivo* under a wide range of effective neutralizing titers. Our results may have implications on the protective effect of neutralizing serum at reduced titers against the antigenic variants of SARS-CoV-2.

## RESULTS

### Antigenic characterization of RBD-binding MAbs.

**FIG 1:**
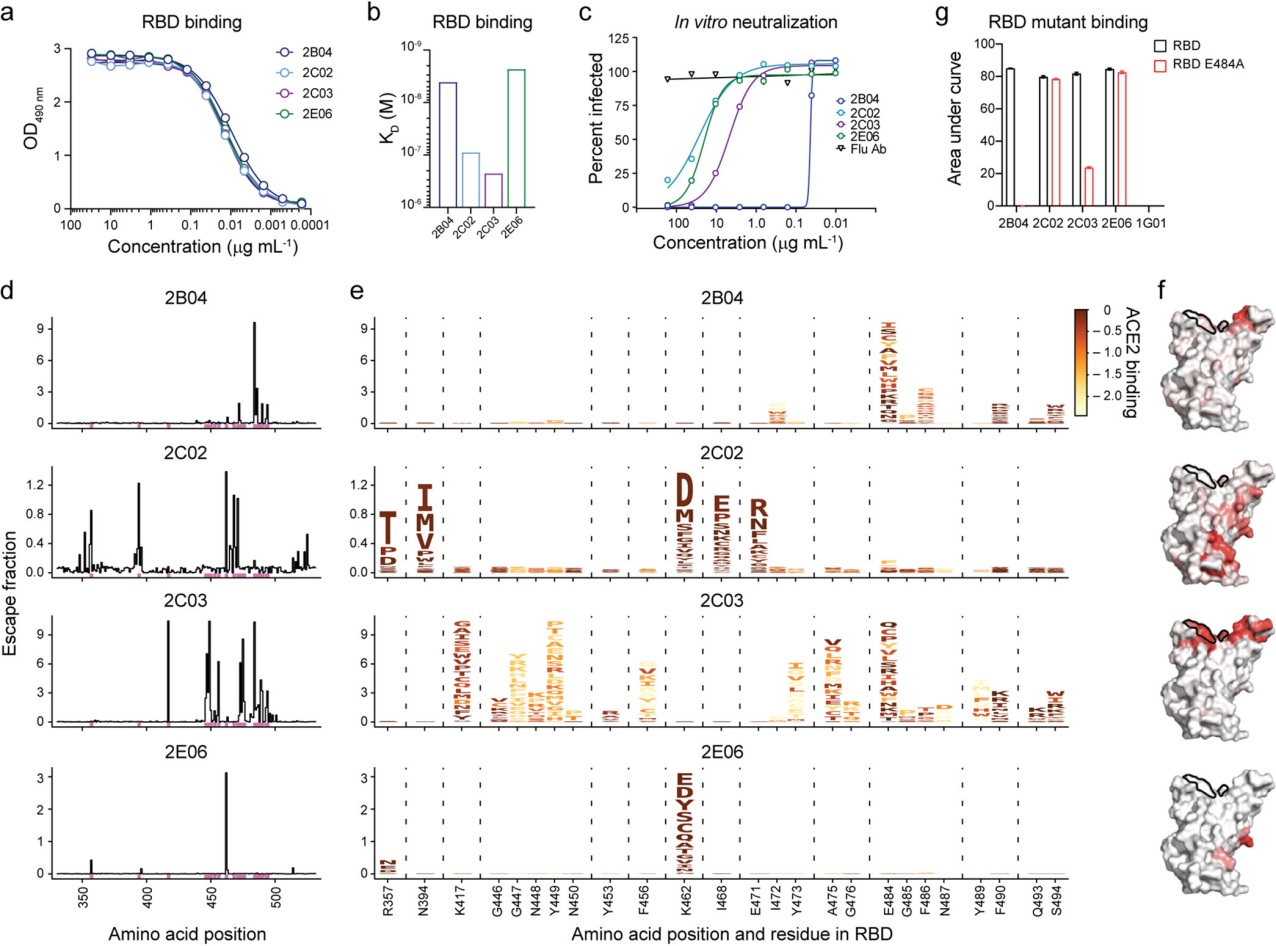
A single amino acid substitution abrogates MAb 2B04 binding to SARS-CoV-2 RBD. (a and b) ELISA binding to recombinant RBD (a) and binding affinity measured by biolayer interferometry (b) of RBD-specific MAbs. OD_490 nm_, optical density at 490 nm. (c) Percent relative infection by chimeric vesicular stomatitis virus harboring the SARS-CoV-2 S protein as a function of MAb concentration in a GFP reduction neutralization test. (d) Mutational antigenic profiling of RBD-specific MAbs. The sum of the escape fractions of all amino acid substitutions at each site in the RBD for each Mab is shown. Sites of strong binding escape for any of the four antibodies are indicated by pink bars along the *x* axis and shown in greater detail in panel e. (e) Logo plots showing the effects of each amino acid substitution on antibody binding, with taller letters indicating more binding escape, and letters colored according to how deleterious mutations are for ACE2 binding (20). Interactive visualizations of escape data can be found at https://jbloomlab.github.io/SARS-CoV-2-RBD_MAP_Ellebedy_Abs/. (f) Antibody escape mapped to the RBD surface (PDB accession no. 6M0J [37]) for 2B04, 2C02, 2C03, and 2E06 MAbs. The ACE2 contact surface is outlined in black. Sites are colored according to the maximum effect of a substitution at a given site, from white (no effect on antibody binding) to red (mutation with the strongest effect on antibody binding). (g) ELISA to measure binding of MAbs to wild-type (black) and E484A mutant (red) RBD. Values are means plus standard errors of the means (SEM) (error bars).

We previously generated and characterized a panel of RBD-binding human chimeric MAbs (human immunoglobulin [hu-Ig]) from mice immunized with recombinant RBD and boosted with S protein of SARS-CoV-2 (18). Four MAbs, 2B04, 2C02, 2C03, and 2E06, that showed similar binding to the RBD of the S protein of SARS-CoV-2 (Fig. 1a), were selected for further characterization. The binding affinity (*K_D_*), measured by biolayer interferometry was <10^−8^ M for MAbs 2B04 and 2E06, whereas the *K_D_* was ≥10^−7^ M for MAbs 2C02 and 2C03 (Fig. 1b). The inhibitory concentration for the MAbs to neutralize 50% of chimeric vesicular stomatitis virus (VSV) expressing the S protein (19) of SARS-CoV-2 (IC_50_) was 0.04 μg/ml, 4.7 μg/ml, 26 μg/ml, and 20 μg/ml for 2B04, 2C03, 2C02, and 2E06, respectively (Fig. 1c), indicating 2B04 is a highly potent neutralizing MAb, 2C03 is a weak neutralizing MAb, and that 2C02 and 2E06 are minimally neutralizing MAbs.

To identify the antibody binding site on the RBD, we generated duplicated mutant yeast display libraries expressing 3,804 of 3,819 possible amino acid substitutions in the RBD derived from the Wuhan-Hu-1 strain of SARS-CoV-2 (20). After eliminating mutants that abrogated ACE2 binding, yeast cells that showed a 100-fold or greater reduction in MAb binding were sorted and sequenced by next-generation sequencing (see Fig. S1a and b in the supplemental material) (21). The frequency of each amino acid substitution was quantified in the pre- and postsorted yeast cells and used to calculate an escape fraction score. The amino acid substitution and location in the RBD domain or S protein are shown in Fig. 1d to f. The predicted epitope for the highly potent MAb 2B04 was located along the receptor binding “ridge” and amino acid changes at a small number of residues, most prominently positions 484, 486, and 494, are predicted to abrogate binding. In contrast, the predicted epitope for the weakly neutralizing MAb 2C03 spans a wider swath of the ACE2-binding surface of S protein. Amino acid changes at many different residues of the RBD reduced 2C03 binding. Many of them would also diminish ACE2 binding and thus are predicted to affect viral fitness (20). The predicted epitope for the nonneutralizing MAb 2C02 was along the “edge” of the core RBD and overlapped with an evolutionarily conserved patch around residue 465. Amino acid changes at five different residues (357, 394, 462, 468, and 471) reduced the binding of the 2C02 MAb. Similar to 2C02, the predicted epitope for the nonneutralizing MAb 2E06 was also within the evolutionarily conserved patch around residue 465. Loss-of-binding mutations for 2E06 were highly focused on residue 462 and residue 357 where a putative glycan knock-in mutation R357N was noted (Fig. 1d and e). To validate the yeast display assay, we generated recombinant RBD with an E484A mutation and measured binding of each MAb to wild type (WT) and E484A RBD by an enzyme-linked immunosorbent assay (ELISA) (Fig. 1g and Fig. S1d). As predicted by the yeast display escape profiles, the E484A mutation completely abolished binding of 2B04, diminished binding by 2C03, but had no effect on the binding of 2C02 or 2E06. Taken together, we observed that the two MAbs with a binding affinity to RBD of <10^−8^ M (2B04 and 2E06) demonstrated focused escape profiles with relatively few residues on the RBD, while MAbs showing weaker binding affinities to RBD (≥10^−7^ M, 2C02 and 2C03) tend to be escaped by mutations at more residues on the RBD.

**FIG 2:**
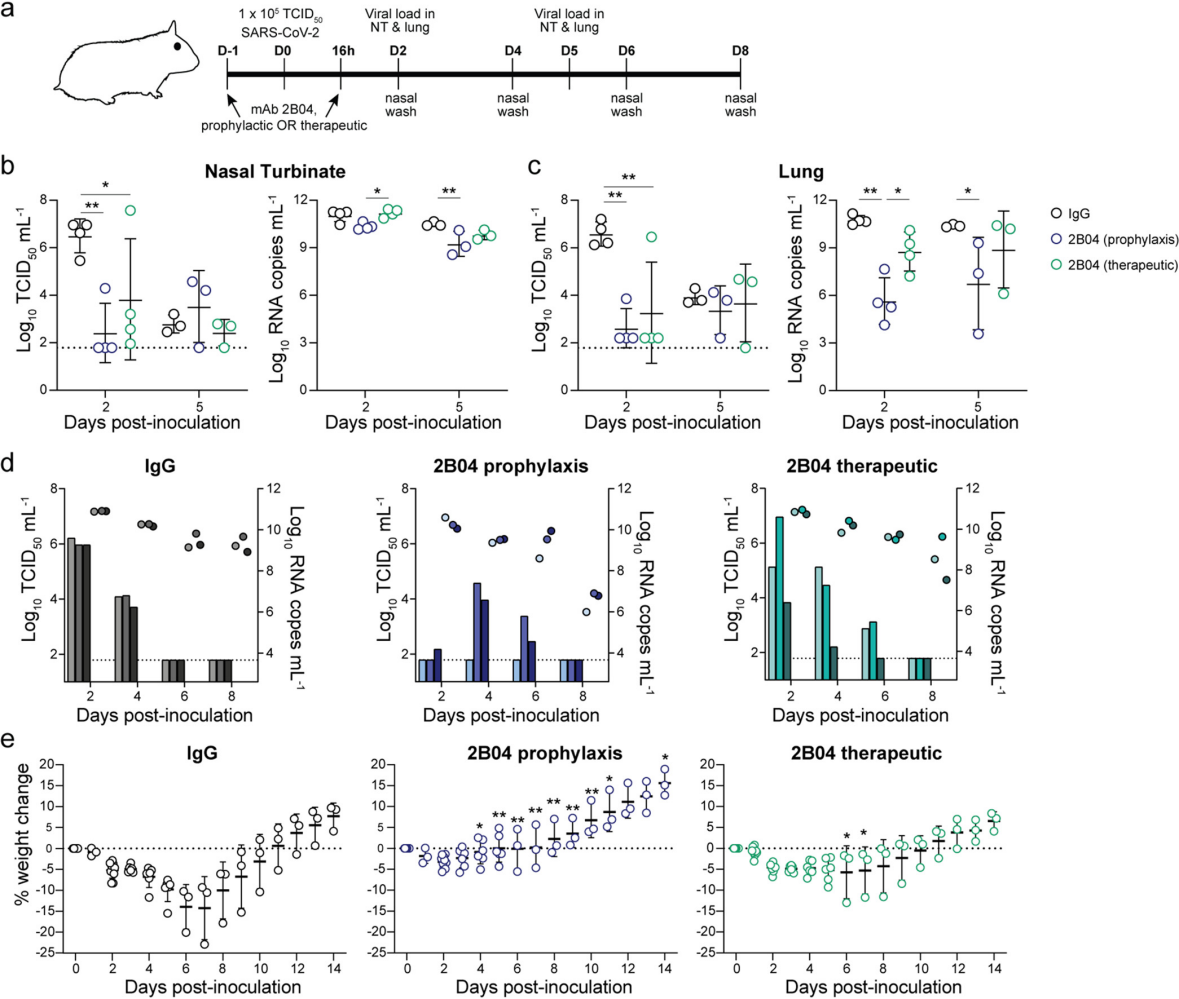
Neutralizing MAb 2B04 protects Syrian hamsters against SARS-CoV-2. (a) Experimental scheme for prophylactic or therapeutic treatment of Syrian hamsters with chimeric human MAb. Hamsters received 1 mg of hu-Ig 2B04 or isotype control (IgG) 24 h prior to (prophylactic) or 16 h after (therapeutic) IN challenge with 10^5^ TCID_50_ of SARS-CoV-2. D-1, day −1; NT, nasal turbinate. (b and c) Infectious virus titer (left) and viral RNA level (right) detected in nasal turbinates (b) and lungs (c). Statistical significance by two-way ANOVA followed by Tukey’s multiple-comparison test is indicated by asterisks as follows: *, *P* < 0.05; **, *P* < 0.01. (d) Infectious virus titer (bars) and viral RNA level (circles) detected in nasal washes on the indicated day postinoculation (dpi). Paired measurements share colors. (e) Percent weight change of hamsters receiving isotype control (left), hu-Ig 2B04 prophylactically (center), or therapeutically (right). Statistical significance by ANOVA with Holm-Sidak correction for multiple comparisons is indicated by asterisks as follows: *, *P* < 0.05, **, *P* < 0.01. Lines indicate means ± standard deviations (SD). The dotted lines in panels b to d show the limit of detection of the assay, and each symbol represents the value for one hamster. The dotted line in panel e represents no weight loss, and each symbol represents the value for one hamster.

10.1128/mBio.02395-21.1 FIG S1 Epitope mapping by mutational antigenic profiling of RBD-specific MAbs. (a) Fluorescence-activated cell sorting (FACS) gates used to select single cell yeast expressing the RBD (FITC-Myc^+^) of the SARS-CoV-2 S protein. Representative images are shown. (b) FACS plots and gating strategy to isolate yeast cell population that do not bind to MAb. Left panels demonstrated yeast cells expressing wild-type RBD detected with 1× or 0.01× MAb concentration. The right two panels demonstrate MAb binding (1× concentration) to yeast cells expressing the RBD mutant libraries. The FACS gates identify yeast cells expressing a loss-of-binding mutant RBD. (c) Correlations in escape fraction scores between replicate libraries. Top plots are the correlation in the per-mutant escape fraction scores (that is, the height of a letter in the logo plots in Fig. 2e). Bottom plots are the correlation in the sum-per-site escape fraction scores (that is, the height of a “stack” of letters in the logo plots). (d) ELISA to measure binding of the four different MAbs to wild-type (black) and E484A mutant (red) RBD. Download FIG S1, TIF file, 1.3 MB. Copyright © 2021 Su et al. 2021 Su et al. https://creativecommons.org/licenses/by/4.0/ This content is distributed under the terms of the Creative Commons Attribution 4.0 International license.

### Prophylactic and therapeutic treatment with 2B04 protects Syrian hamsters against SARS-CoV-2 challenge.

We first evaluated the prophylactic and therapeutic efficacy of the mouse-human chimeric MAb 2B04 (hu-Ig 2B04) against SARS-CoV-2 in 4- to 6-week-old male Syrian hamsters. hu-Ig 2B04 was administered via intraperitoneal (IP) injection 24 h prior to (prophylactic treatment) or 16 h after (therapeutic treatment) intranasal (IN) challenge with 10^5^ 50% tissue culture infective doses (TCID_50_) of SARS-CoV-2 (strain BetaCoV/Hong Kong/VM20001061/2020) (Fig. 2a). Age- and sex-matched control animals received human IgG isotype control antibody IP 24 h prior to SARS-CoV-2 challenge. Prophylactic treatment with hu-Ig 2B04 significantly reduced infectious virus titer by more than 100-fold in the nasal turbinates (*P* < 0.01, *d *=* *5.8) (Fig. 2b) and in the lungs (*P* < 0.01, *d* = 4.8 for prophylactic, and *P* < 0.01, *d *=* *2.1 for therapeutic treatment) (Fig. 2c) on 2 days postinoculation (dpi). Prophylactic treatment also significantly reduced RNA levels detected in the lungs on 2 dpi (*P* < 0.01, *d *=* *4.0) (Fig. 2c). On 5 dpi, the infectious virus titers detected in the nasal turbinates and lungs were comparable between the isotype control antibody-treated and hu-Ig 2B04-treated animals (Fig. 2b and c). However, the viral RNA level was reduced in the nasal turbinates (*P* < 0.01, *d *=* *2.2) and in the lungs (*P* < 0.05, *d *=* *1.8) of animals that received prophylactic hu-Ig 2B04 treatment. Prophylactic treatment (*P* < 0.0001, *d *=* *22.1), but not therapeutic treatment (*P* = 0.53), with hu-Ig 2B04 reduced infectious virus titers in the nasal washes on 2 dpi compared to the isotype control group (Fig. 2d); there was no significant difference in infectious virus load detected on 4, 6, or 8 dpi or by comparing the total area under the curve. The reduced viral load is reflected in the weight changes of the animals, as animals treated with hu-Ig 2B04 prophylactically or therapeutically lost significantly less weight than those that received isotype control antibody (Fig. 2e). The maximal average weight loss for the isotype control antibody, hu-Ig 2B04 prophylactic treatment, and hu-Ig 2B04 therapeutic treatment groups were 14.3% (on 7 dpi), 3.3% (on 2 dpi), and 5.7% (on 6 dpi), respectively. Overall, prophylactic and to a lesser extent therapeutic treatment with hu-Ig 2B04 showed substantial capacity to decrease the viral load in upper and lower respiratory tissues upon SARS-CoV-2 challenge.

### Hamster Fc 2B04 MAb protects Syrian hamsters against SARS-CoV-2 challenge.

**FIG 3:**
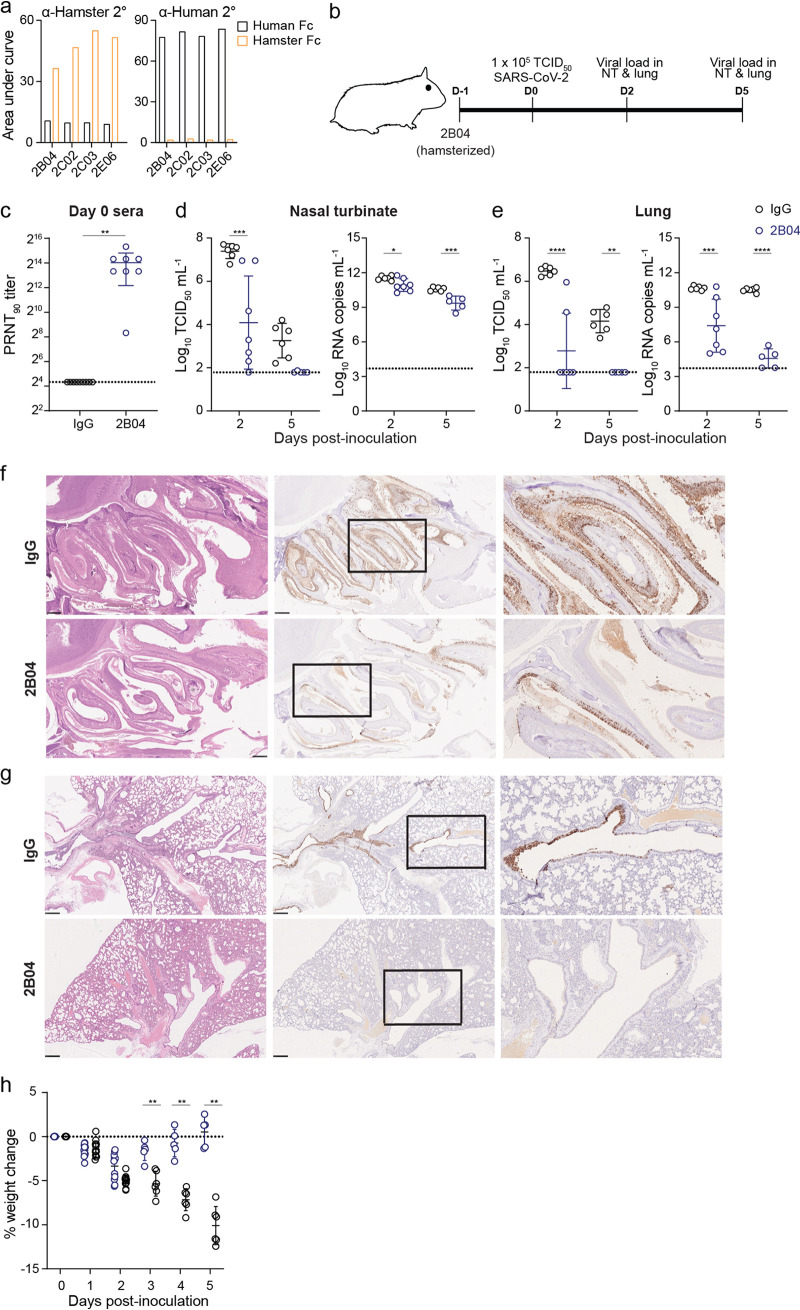
Chimeric hamster neutralizing MAb protects Syrian hamsters against SARS-CoV-2. (a) Detection of RBD-specific chimeric human (black) or hamster (orange) MAbs with anti-hamster (α-hamster) or anti-human secondary (2°) antibodies, respectively. (b) Experimental scheme for SARS-CoV-2 challenge of Syrian hamsters receiving 1 mg of chimeric hamster 2B04 (ham-Ig) or isotype control 24 h prior to IN challenge with 10^5^ TCID_50_ of SARS-CoV-2. (c) Serum virus neutralizing titer (PRNT_90_) 1 day after IP administration of 1 mg of chimeric hamster 2B04 or isotype control (IgG). (d and e) Infectious virus titer (left) and viral RNA copy numbers (right) detected in nasal turbinate (d) and lungs (e) 2 and 5 dpi. Lines indicate mean ± SD. The dotted lines in panels c to e show the limit of detection of the assay, and each symbol represents the value for one hamster. Statistical significance by two-way ANOVA followed by Tukey’s multiple-comparison test is indicated by asterisks as follows: *, *P* < 0.05, **, *P* < 0.01, ***, *P* < 0.001, ****, *P* < 0.0001. (f and g) Hematoxylin and eosin staining (left panels) and immunohistochemistry for SARS-CoV-2 N protein (brown color, middle and right panels) in nasal turbinates (f) and lungs (g) of SARS-CoV-2-challenged hamsters 2 dpi. Scale bars = 500 μm. Higher-magnification images (right panels) of the boxed areas in the immunochemistry sections (middle panels) are shown for lungs and nasal turbinates. (h) Weight changes of hamsters receiving isotype (IgG, black circles) or 2B04 (blue circles) after SARS-CoV-2 challenge. Statistical significance by two-way ANOVA with Holm-Sidak correction for multiple comparisons is indicated by asterisks as follows: **, *P* < 0.01; ****, *P* < 0.0001. The dotted line in panel h represents no weight loss and each symbol represents one hamster.

Since the human Fc portion of the hu-Ig 2B04 may not mediate Fc-mediated effector function in the Syrian hamster model, the murine variable heavy and light chain of MAb 2B04, were cloned into expression vectors coding for hamster IgG2a constant regions to produce chimeric hamster MAbs (ham-Ig 2B04 [Fig. S2a]). We confirmed that the ham-Ig 2B04 MAb reacted with anti-hamster but not with anti-human secondary antibodies (Fig. 3a). Prophylactic efficacy of ham-Ig 2B04 compared to hamster IgG2a isotype control was evaluated in groups of hamsters treated 24 h prior to IN challenge with 10^5^ TCID_50_ of SARS-CoV-2 (Fig. 3b). Sera collected from eight hamsters 1 day after IP injection of ham-Ig 2B04 showed an average 90% plaque reduction neutralization titer (PRNT_90_) titer of 1:10,200 (Fig. 3c). Compared to the isotype control group, prophylactic treatment with ham-Ig 2B04 reduced the average infectious virus titer by >3.0 log_10_ units in the nasal turbinates on 2 dpi (*P* < 0.001, *d *=* *2.1). The infectious virus titer in the nasal turbinates was comparable on 5 dpi (*P* = 0.10) (Fig. 3d). The viral RNA levels in the nasal turbinates were significantly lower between the ham-Ig 2B04 and control antibody-treated animals 2 dpi (*P* < 0.05, *d *=* *1.5) and 5 dpi (*P* < 0.001, *d *=* *3.0). In the lungs, prophylactic treatment with ham-Ig 2B04 reduced the mean infectious virus titer by 3.7 and 2.4 log_10_ units compared to the isotype control antibody-treated animals on 2 dpi (*P* < 0.0001, *d *=* *3.0) and 5 dpi (*P* < 0.01, *d *=* *6.3), respectively (Fig. 3e). Viral RNA levels were similarly reduced by 1,700- and 800,000-fold in the lungs of ham-Ig 2B04-treated animals on 2 dpi (*P* < 0.001, *d *=* *2.0) and 5 dpi (*P* < 0.0001, *d *=* *9.8), respectively. To support these findings, immunohistochemistry was performed on formalin-fixed nasal turbinates and lungs from ham-Ig 2B04-treated and control antibody-treated animals. Less SARS-CoV-2 antigen was detected in the nasal turbinates (Fig. 3f) or lungs (Fig. 3g) of the ham-Ig 2B04-treated hamsters compared to the isotype control antibody-treated hamsters. Finally, the maximal average weight losses for the isotype control antibody- and the ham-Ig 2B04-treated animals were 10.1% (on 5 dpi) and 3.4% (on 2 dpi), respectively (Fig. 3h). Starting 3 dpi, the ham-Ig 2B04-treated animals lose significantly less weight (*P* < 0.001 for 3 dpi and *P* < 0.0001 for 4 and 5 dpi) compared to the isotype-treated animals. The results showed a comparable protective effect between hu-Ig 2B04 and ham-Ig 2B04 against SARS-CoV-2 in the hamster model. Importantly, no deleterious effect was observed in hamsters after receiving a chimeric hamster MAb for prophylactic treatment.

10.1128/mBio.02395-21.2 FIG S2 Generation of hamster Fc-bearing MAbs. (a) Schematic of mouse-human (hu-Ig) and mouse-hamster (ham-Ig) chimeric MAbs as originally generated (18) and used in Fig. 1, 2, 3, and 5, respectively. (b to d) Alignment of immunoglobulin constant regions for the heavy chain (IGHG [b]), kappa light chain (IGKC [c]). and lambda light chain (IGLC [d]) of human (Homo sapiens [Hs]), Armenian hamster (Cricetulus migratorius [Cm]), and Syrian hamster (Mesocricetus auratus [Ma]). In panel d, mouse-derived residues 1 to 13 from original cloning from mouse plasmablasts are boxed. Download FIG S2, TIF file, 1.2 MB Copyright © 2021 Su et al. 2021 Su et al. https://creativecommons.org/licenses/by/4.0/ This content is distributed under the terms of the Creative Commons Attribution 4.0 International license.

### Hamster Fc 2B04 MAb prevented SARS-CoV-2 aerosol transmission in hamsters.

**FIG 4:**
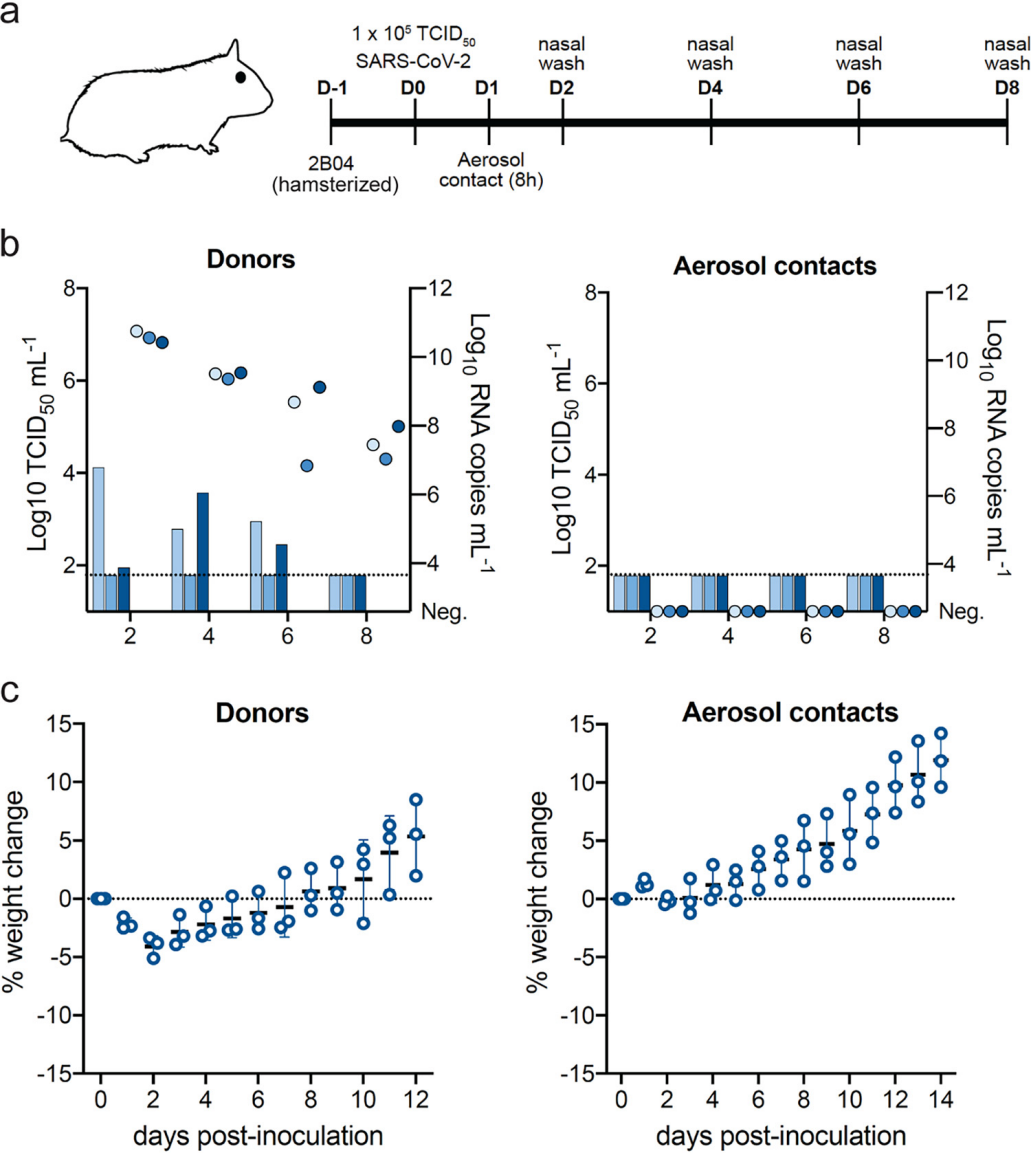
Chimeric hamster neutralizing MAb protect against SARS-CoV-2 transmission. (a) Experimental scheme for SARS-CoV-2 challenge and aerosol transmission in Syrian hamsters receiving 1 mg of ham-Ig 2B04. (b) Infectious virus titer (bars) and viral RNA copy numbers (circles) detected in nasal washes of inoculated donor animals (left) and the aerosol-contact animals (right). (c) Weight changes of donor (left) and aerosol-contact (right) hamsters. The dotted line in panel b shows the limit of detection of the assay, and each symbol represents the value for one hamster. The dotted line in panel c represents no weight loss, and each symbol represents the value for one hamster.

We have demonstrated previously that SARS-CoV-2 transmits efficiently among hamsters via respiratory aerosols (22). In this experimental model, naive hamsters that were exposed to SARS-CoV-2-inoculated donors on 1 dpi for 8 h were 100% infected (in three pairs of donor and aerosol-contact hamsters), shed infectious viruses for 5 days after exposure (Fig. 3d of reference 22), and with 7.72% maximal loss on day 7 postexposure (Fig. 3f of reference 22). Since prophylactic treatment of ham-Ig 2B04 reduced viral loads in the nasal turbinates and lungs on 2 dpi (Fig. 3d to g), we further evaluated whether prophylactic treatment with ham-Ig 2B04 in donor hamsters reduced SARS-CoV-2 transmission to naive untreated hamsters. We followed the identical experimental design as reported previously (22), except that the donor hamsters received ham-Ig 2B04 24 h prior to IN challenge with 10^5^ TCID_50_ of SARS-CoV-2 (Fig. 4a). Nasal washes collected on 2, 4, 6, and 8 dpi of the donors (1, 3, 5, and 7 days postexposure of the aerosol contacts) showed reduced viral replication after 2B04 prophylactic treatment, with no virus shedding in one animal and reduced infectious virus titers detected in the other two animals (Fig. 4b). Importantly, no infectious virus was detected from any of the aerosol-contact hamsters (Fig. 4b) and none of the aerosol-contact animals developed neutralizing antibodies against SARS-CoV-2 at 14 days postexposure (serum PRNT_90_ of <1:20). No apparent weight loss was observed from aerosol-contact animals (Fig. 4c). These results suggest that potent neutralizing antibodies may reduce viral load after SARS-CoV-2 infection and block onward transmission via aerosols to naive animals. Furthermore, histopathological examination of tissues (nasal, lung, heart, liver, spleen, and kidney) collected on day 12 postchallenge of SARS-CoV-2 from donor hamsters treated prophylactically with ham-Ig 2B04 found no viral antigen, inflammation, or infiltration of immune cells associated with immunopathology (data not shown).

### Efficacy of weak neutralizing and nonneutralizing antibodies against SARS-CoV-2 in Syrian hamsters.

**FIG 5:**
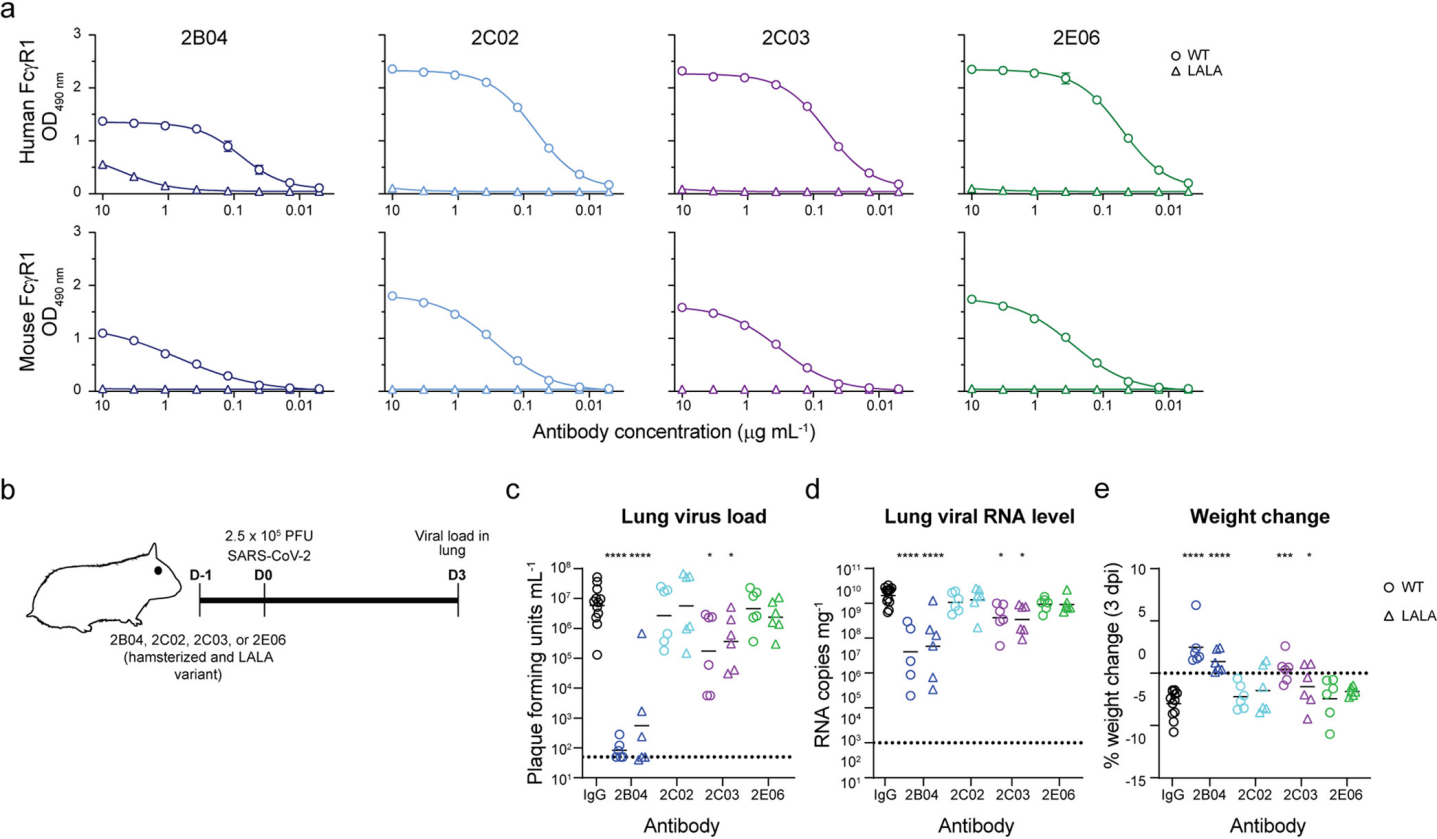
Neutralizing MAbs protect Syrian hamsters from SARS-CoV-2 infection in a Fc-independent manner. (a) ELISA binding of wild-type (circles) and LALA mutant (triangles) chimeric hamster MAbs to human (top panels) and mouse (bottom panels) FcγR1. (b) Experimental scheme for SARS-CoV-2 challenge of hamsters receiving 1 mg of wild-type or LALA mutant ham-Ig 2B04, 2C02, 2C03, or 2E06 24 h prior to intranasal challenge with 2.5 × 10^5^ PFU of SARS-CoV-2. (c) Infectious virus titer in the lungs of isotype or wild-type and LALA mutant ham-Ig 2B04-, 2C02-, 2C03-, or 2E06-treated hamsters. Statistical significance by Kruskal-Wallis test with a multiple comparison correction between isotype and wild- type or LALA 2B04-, 2C02-, 2C03-, and 2E06-treated groups is indicated by asterisks as follows: *, *P* < 0.05; ****, *P* < 0.0001. Lines represent geometric mean ± geometric SD, and the dotted line is the limit of detection for the assay. Each symbol represents the value for one hamster. (d) Viral RNA copy numbers in lungs of isotype or wild-type and LALA mutant ham-Ig 2B04-, 2C02-, 2C03-, or 2E06-treated hamsters. Statistical significance by one-way ANOVA and a multiple comparison correction between isotype and wild type or LALA 2B04-, 2C02-, 2C03-, and 2E06-treated groups is indicated by asterisks as follows: *, *P* < 0.05, ****, *P* < 0.0001. Lines represent the geometric mean, and the dotted line is the limit of detection for the assay. Each symbol represents the value for one hamster. (e) Weight changes of hamsters receiving the indicated wild-type (WT) (circles) or LALA mutant (triangles) MAb 3 dpi. Statistical significance by one-way ANOVA with a multiple comparisons between isotype and wild-type or LALA 2B04-, 2C02-, 2C03-, and 2E06-treated groups is indicated by asterisks as follows: *, *P* < 0.05; ***, *P* < 0.001; ****, *P* < 0.0001. The dotted line represents no weight loss. Each symbol represents the value for one hamster.

Thus far, we have demonstrated the protective effect of a highly potent neutralizing MAb, 2B04, against SARS-CoV-2 challenge in hamsters. Next, we evaluated a weakly neutralizing 2C03 that targets an epitope overlapping that of 2B04, and two minimally neutralizing MAbs (2C02 and 2E06) that target a unique epitope in the core RBD but exhibit different binding affinities *in vitro* (Fig. 1a). To optimize Fc-Fc receptor interactions in the hamsters, all MAbs were expressed as mouse-hamster chimeric MAbs (ham-Ig [Fig. S2]). To further delineate whether the protective mechanisms conferred by these ham-Ig MAbs was dependent on Fc receptor binding, we generated ham-Ig MAbs bearing two leucine-to-alanine substitutions at positions 123 and 124 (LALA). To confirm that the LALA substitutions abrogated Fc receptor binding, a capture ELISA, using recombinant human and mouse FcγR1 chain (CD64) was performed. The ham-Ig 2B04, 2C02, 2C03, and 2E06 all bound to the human and mouse FcγR1 chain, albeit the binding was lower for the murine FcγR1 receptor. Importantly, the LALA substitutions completely abrogated binding of the ham-Ig MAbs to both human and mouse FcγR1 (Fig. 5a and Fig. S3). To test the protective efficacy of these antibodies and the role of Fc receptor engagement, groups of 5- to 6-week-old male hamsters received either the wild-type or LALA mutant of ham-Ig 2B04, 2C02, 2C03, or 2E06 24 h prior to IN inoculation with 2.5 × 10^5^ PFU of SARS-CoV-2 virus (Fig. 5b). Infectious virus titer, viral RNA level, and weight loss were measured on 3 dpi. Both the highly potent neutralizing ham-Ig 2B04 (*P* < 0.0001, *d *=* *9.1) and the weakly neutralizing ham-Ig 2C03 (*P* < 0.05, *d *=* *1.4) significantly reduced infectious virus titers in the lungs on 3 dpi (Fig. 5c). This reduction corresponded to lower viral RNA levels (*P* < 0.0001 and *d *=* *2.5 for 2B04; *P* < 0.05 and *d *=* *1.7 for 2C03) and less weight loss (*P* < 0.0001 and *d *=* *3.2 for 2B04; *P* < 0.001 and *d *=* *2.5 for 2C03) (Fig. 5d and e). Furthermore, the wild-type and LALA versions of 2B04 and 2C03 showed comparable effects in reducing lung viral load (*P* < 0.0001 and *d *=* *3.2 for 2B04; *P* < 0.05 and *d *=* *1.5 for 2C03 [Fig. 5c]), viral RNA copy number (*P* < 0.0001 and *d *=* *2.1 for 2B04; *P* < 0.05 and *d *=* *1.9 for 2C03 [Fig. 5d]), and preventing weight loss (*P* < 0.0001 and *d *=* *3.4 for 2B04; *P* < 0.05 and *d *=* *0.9 for 2C03 [Fig. 5e]), suggesting that the protection was mediated in an Fc receptor-independent manner. In contrast, neither ham-Ig 2C02 nor ham-Ig 2E06 significantly reduced infectious virus titers or viral RNA levels in the lungs 3 dpi (Fig. 5c and d). Also, treatment of ham-Ig 2C02 or ham-Ig 2E06 did not prevent weight loss in hamsters challenged with SARS-CoV-2. These results suggest that prophylactic treatment with weakly neutralizing antibodies confer some protection from SARS-CoV-2 infection if targeting particular epitopes and that nonneutralizing MAbs do not enhance infection. We also demonstrated that the highly potent and weak neutralizing antibodies targeting the RBD of S protein conferred protection against SARS-CoV-2 infection in an Fc-independent manner.

10.1128/mBio.02395-21.3 FIG S3 Fc receptor binding by LALA mutants. ELISA binding of indicated wild-type (circles) and LALA mutant (triangles) mouse-hamster chimeric MAbs to mouse FcγRIIb (top panels) and mouse FcγRIII (bottom panels). Download FIG S3, TIF file, 0.2 MB. Copyright © 2021 Su et al. 2021 Su et al. https://creativecommons.org/licenses/by/4.0/ This content is distributed under the terms of the Creative Commons Attribution 4.0 International license.

## DISCUSSION

Understanding how antibodies confer protection against SARS-CoV-2 *in vivo* is essential for the development and implementation of antibody therapy and vaccines. Specifically, the role of poorly neutralizing or nonneutralizing antibodies targeting the RBD of the S protein of SARS-CoV-2 on protection against COVID-19 is not well understood. In this study, we antigenically characterized four MAbs with different virus neutralizing capacities and evaluated their efficacy in the SARS-CoV-2 hamster model. A highly potent MAb, 2B04, prevented weight loss in hamsters if given prophylactically or therapeutically; prophylactic treatment also abolished onward transmission to naive and untreated contact animals. Interestingly, administration of a weak neutralizing MAb 2C03 prior to SARS-CoV-2 challenge also reduced infectious virus titers and protected against weight loss. Importantly, the protective effect of these neutralizing antibodies was independent of Fc-Fc receptor interactions. Combined, these studies suggest the potential benefit of low levels of neutralizing antibodies against SARS-CoV-2 infection.

Prophylactic treatment of a potent neutralizing MAb effectively reduced viral loads in the nasal tissue and nasal wash of donor hamsters, which blocked SARS-CoV-2 transmission to naive untreated hamsters via respiratory aerosols. This is the first demonstration that parenteral delivery of MAb can reduce or eliminate transmission of SARS-CoV-2, and it suggests that a highly potent neutralizing IgG response elicited after vaccination may protect the mucosal surface of the upper airways. The antibody threshold required to protect against onward transmission remains to be investigated. We found that the average serum antibody neutralization titer (PRNT_90_) in ham-Ig 2B04-treated hamsters was ∼1:10,000. This is higher than what is found in the sera of most vaccinated and infected individuals. Also, with the emerging resistance of SARS-CoV-2 variants to antibody neutralization, it is possible that the neutralizing antibody titer in vaccinated individuals is not sufficient to eliminate transmission of SARS-CoV-2 infection.

The four MAbs in this study bind to two antigenically distinct epitopes on the surface of the S protein of SARS-CoV-2. The predicted epitopes for MAbs 2B04 and 2C03 were located in the receptor-binding motif (RBM). 2B04 selects escape mutations localized to the ACE2-binding ridge, similar to MAbs COV2-2832, COV2-2479, and COV2-2050 (21). Coincidentally, amino acid changes between residues 475 to 490 on the RBD significantly impacted the binding of these MAbs to the RBD. The predicted epitope for MAb 2C03 overlaps the ACE2-binding ridge sites selected by 2B04 but also spans a broader set of sites across the RBM surface, similar to LY-CoV016 (23). Virus isolates harboring an E484K mutation in the RBD of the S protein have emerged recently (24, 25), and it is likely that this mutation will escape binding and neutralization by these two MAbs. The two minimally neutralizing antibodies, 2C02 and 2E06, recognize an epitope along the “edge” of the core RBD and overlap with an evolutionarily conserved patch around residue 465. To our knowledge, this is the first description of an antibody that binds this epitope (9). Interestingly, these two antibodies cross-reacted with the S protein of SARS-CoV-1 (18). Antigenic mapping of other SARS-CoV-1 cross-reactive antibodies (CR3022, COV2-2082, and COV2-2094) (21) revealed that these antibodies also target the core of the RBD; however, the predicted epitope is distinct from that of 2C02 and 2E06. Besides the location of the predicted epitope, the affinity of the neutralizing (2B04 and 2C03) and minimally neutralizing (2C02 and 2E06) antibodies were distinctly different. Additional studies are needed to determine the significance of these two variables on the overall effectiveness of anti-SARS-CoV-2 antibodies.

Fc-Fc receptor interactions were not required when Mab 2B04 and 2C03 were administered prior to SARS-CoV-2 exposure, suggesting their mechanism of protection depends largely on the neutralizing capacity of the antibody to prevent initial viral infection and limit dissemination. This finding is in agreement with previous reports showing that LALA-PG loss-of-function Fc variant of potently neutralizing antibodies were equally effective when administered prophylactically (26, 27). However, the *in vivo* efficacy of LALA-PG antibodies were diminished compared to their WT counterparts when the MAb was administered 1 day after SARS-CoV-2 infection (26, 27). This reduction was associated with increased virus titers, weight loss, and inflammatory gene expression in the LALA-PG-treated animals. The requirement of Fc effector function varied among MAbs (26).

One of the limitations of this study is that we tested the Fc-Fc receptor interaction requirements for a single hamster IgG subclass. Different IgG subclasses have different affinities for Fc receptors, and therefore, it is possible that the LALA mutations differentially affect the IgG subclasses in hamsters. Due to the lack of reagents for hamsters, we also did not evaluate the impact of complement binding or activation of the hamster Fc-containing antibodies. Another limitation of our study is that we did not establish a serological correlate of protection against SARS-CoV-2 infection and transmission. This is a particularly important question given the emergence of SARS-CoV-2 variants and increasing number of breakthrough infections in vaccinated individuals. Given the size and scope of this analysis, we believe that this is a separate study. Finally, we did not include an isotype control in the transmission studies and only compare the results with the untreated donor hamsters (22). We and others have shown that the administration of an isotype antibody has no effect on the infectious virus titers in nasal washes and lungs, and therefore, we believe that the lack of transmission of SARS-CoV-2 in the 2B04-treated animals is due to the neutralization of the virus and not due to the IP administration of a MAb.

Overall, our studies in hamsters demonstrate that potent neutralizing MAbs protect against SARS-CoV-2 transmission and severe disease and that this activity is independent of Fc receptor engagement. The ability of a weakly neutralizing MAb to reduce viral loads in the lungs and protect animals from weight loss suggests that low or reduced neutralizing antibody titers due to waning immunity, subclinical infections, or against antigenic variants may still provide protection from severe disease *in vivo*.

## MATERIALS AND METHODS

### Cells and viruses.

Expi293F cells (Gibco) were cultured at 37°C and 8% CO_2_ in Expi293 expression medium (Gibco) with 130 rpm shaking. Vero E6 cells (CRL-1586; ATCC), Vero CCL81 cells (ATCC), Vero-Creanga cells (Vero cells overexpressing human ACE2 and TMPRSS2, gift from Adrian Creanga and Barney Graham, NIH), and HEK293 cells were cultured at 37°C in Dulbecco’s modified Eagle medium (DMEM) supplemented with 10% fetal bovine serum (FBS), 10 mM HEPES (pH 7.3), 1.0 mM sodium pyruvate, 1× nonessential amino acids, and 100 U/ml of penicillin-streptomycin. MA-104 cells (CRL-2378; ATCC) were cultured at 37°C in 199 medium supplemented with 5% FBS and 2.5 μg/ml of amphotericin B.

SARS-CoV-2 (strain BetaCoV/Hong Kong/VM20001061/2020) was expanded three times in Vero E6 cells in DMEM supplemented with 4.5 g/liter d-glucose, 100 mg/liter sodium pyruvate, 2% FBS, 100 U/ml penicillin-streptomycin, and 25 mM HEPES. The consensus sequence of the expanded stock virus (10^7.25^ TCID_50_/ml) was identical to the original specimen (BioProject accession no. PRJNA741371). SARS-CoV-2 (strain 2019-nCoV/USA-WA1/2020) was obtained from the U.S. Centers for Disease Control (CDC) and propagated on MA-104 monkey kidney cells. The virus stock was sequenced by next-generation sequencing, and the spike protein sequence was identical to the original WA1 isolate. However, approximately 50% of the sequences, contained a 30- to 36-nucleotide deletion at the furin cleavage of the spike protein. The virus titer of this stock was determined by a focus-forming assay (6.9 × 10^4^ focus-forming units/ml [ffu/ml]) and plaque assay (5.2 × 10^6^ plaque-forming units/ml [PFU/ml]).

### Recombinant proteins.

The receptor binding domain (RBD) of the spike protein of SARS-CoV-2 was generated as previously described (28). Mammalian cell codon-optimized nucleotide sequences coding for the wild type and an E484A mutant (generated using site-directed mutagenesis [QuikChange Lightning; Agilent]) of the receptor binding domain (RBD, amino acids 319 to 541) along with the signal peptide (amino acids 1 to 14) and a hexahistidine tag were cloned into mammalian expression vector pCAGGS. Recombinant proteins were produced in Expi293F cells (ThermoFisher) by transfection with purified DNA using the ExpiFectamine 293 transfection kit (ThermoFisher). Supernatants from transfected cells were harvested 3 days posttransfection, and recombinant RBD was purified using nickel-nitrilotriacetic acid (Ni-NTA) agarose (ThermoFisher), then buffer exchanged into phosphate-buffered saline (PBS), and concentrated using Amicon Ultracel centrifugal filters (EMD Millipore).

### Monoclonal antibodies.

Chimeric mouse-human SARS-CoV-2 RBD-specific MAbs (hu-Ig) 2B04, 2C02, 2C03, and 2E06 were described previously (18). To generate hamster Fc-bearing variants (ham-Ig), their V-J coding segments were cloned into vectors coding for hamster IgG2a, heavy and light chain constant regions (see Fig. S2 in the supplemental material). The immunoglobulin constant gene coding regions for IGHG2A-like and Kappa were derived from previously identified Armenian hamster (Cricetulus migratorius) mRNAs (GenBank accession no. U17166.1 and S80615.1) (29, 30). The coding region for the hamster lambda constant is the predicted annotation “immunoglobulin lambda-1 light chain-like” (RefSeq accession no. XM_021231508.1, LOC101839749) from Syrian hamster (Mesocricetus auratus) genomic DNA (BioProject accession no. PRJNA210213). Antibody V-J coding segments were either synthesized in-frame with the constant regions (2B04, 2C02, and 2C03) along with restriction enzyme sites in the same locations as the pAbVec6W vector (31), or V-J regions were cloned into the hamster vector backbones by standard restriction endonuclease subcloning. To generate LALA mutants, the leucine residues for the LALA mutation in the hamster Fcγ gene, positions 123 and 124, were identified by alignment with the human gamma Fc gene. The LALA mutations in the hamster Fcγ expression vectors were introduced by site-directed mutagenesis (QuikChange Lightning; Agilent).

### SARS-CoV-2 hamster studies (The University of Hong Kong [HKU]).

All procedures involving animals were performed in accordance with guidelines of the Committee on the Use of Live Animals in Teaching and Research, The University of Hong Kong (CULATR no. 5411-20). Four- to 6-week-old male AURA Syrian hamsters were obtained from Laboratory Animal Services Centre, The Chinese University of Hong Kong and housed in the biosafety level 3 (BSL-3) core facility, Li Ka Shing Faculty of Medicine, The University of Hong Kong. Animals were randomized from different litters into experimental groups and were acclimatized at the BSL-3 facilities for 4 to 6 days prior to experiments. Hamsters received 1 mg of isotype control or anti-SARS-CoV-2 MAbs via intraperitoneal (IP) injection. For hamsters receiving ham-Ig 2B04 prophylactically, the median weight was 93.22 g (interquartile range, 90.10 to 96.78 g), and the median MAb dose received was 10.73 mg/kg of body weight (interquartile range, 10.33 to 11.10 mg/kg). Injections were given either at 24 h prior to (prophylactic regimen) or at 16 h after (treatment regimen) SARS-CoV-2 challenge. For challenge studies, hamsters were anesthetized with ketamine (150 mg/kg) and xylazine (10 mg/kg) via IP injection and were intranasally (IN) inoculated with 1 × 10^5^ TCID_50_ of SARS-CoV-2 in 80 μl DMEM (Fig. 2 to 4). Animal weights were measured every day for the duration of experiments. Nasal washes were collected 2, 4, 6, and 8 days postinoculation (dpi). For nasal wash collection, hamsters were anesthetized using ketamine (100 mg/kg) and xylazine (10 mg/kg) via IP injection, and 160 μl PBS containing 0.3% bovine serum albumin (BSA) was used to collect nasal washes from both nostrils of each hamster. Nasal washes were diluted 1:1 by volume and aliquoted for TCID_50_ assay in Vero E6 cells and for quantitative reverse transcription-PCR (RT-PCR). Animals were euthanized 2, 3, or 5 dpi, and the nasal turbinates and lungs were collected for virological or histological analyses. Left lung lobes were homogenized in 1 ml of PBS or DMEM, clarified by centrifugation, and used for virus titer determination.

To evaluate the effect of MAb treatment on SARS-CoV-2 transmission via aerosols, we adopted the identical experimental design as reported previously, including the stock virus, dose of inoculation for the donor hamsters, and the exposure time (22). In brief, one naive hamster was exposed for 8 h to one inoculated donor hamster in two adjacent stainless steel wired cages at 1 dpi of the donor animal. The donor animals were injected 24 h prior to SARS-CoV-2 infection with 1 mg of isotype control or anti-SARS-CoV-2 MAb via IP injection. DietGel 76A (ClearH_2_O) was provided to the hamsters during the 8-h exposure. Exposure was done by holding the hamsters inside individually ventilated cages (IsoCage N; Techniplast) with 70 air changes per h. Experiments were repeated with three pairs of donor and aerosol-contact hamsters at a 1:1 ratio. After exposure, the hamsters were housed singly in separate cages and were monitored daily for 14 days. Nasal washes were collected 2, 4, 6, and 8 dpi, and infectious virus titers and viral RNA loads were quantified.

### SARS-CoV-2 hamster studies (Washington University).

All procedures involving animals were performed in accordance with guidelines of the Institutional Animal Care and Use Committee of Washington University in Saint Louis. Five-week-old male hamsters were obtained from Charles River Laboratories and housed in the enhanced BSL-3 facility at Washington University. The animals were acclimatized for 5 or 6 days prior to experiments. Hamsters received 1 mg of isotype control or anti-SARS-CoV-2 MAbs via intraperitoneal (IP) injection 24 h prior to challenge. Following sedation with isoflurane, the animals were challenged via the IN route with 2.5 × 10^5^ PFU of SARS-CoV-2 (Fig. 5). Animal weights were measured daily for the duration of the experiment. Three days after challenge, the animals were sacrificed, and their lungs were collected for virological analysis. The left lobe was homogenized in 1.0 ml DMEM, clarified by centrifugation (1,000 × *g* for 5 min) and used for viral titer analysis by quantitative RT-PCR using primers and probes targeting the N gene, and by a focus-forming assay (FFA).

### Plaque reduction neutralization titer assay.

Sera collected from hamsters 24 h after 2B04 antibody injection were heat inactivated at 56°C for 30 min, serially diluted, and incubated with 30 to 40 PFU of SARS-CoV-2 for 1 h at 37°C. The virus–serum mixtures were added to Vero E6 cells seeded in 24-well culture plates and incubated 1 h at 37°C and 5% CO_2_. The plates were overlaid with 1% agarose in cell culture medium and incubated for 3 days. Thereafter, the plates were fixed with 10% formalin in PBS and stained with 0.3% crystal violet in PBS. Antibody neutralization titers were defined as the highest serum dilution that resulted in >90% reduction in the number of plaques (PRNT_90_).

### Virus neutralization assay with chimeric VSV-S.

Neutralization assays with chimeric VSV expressing the SARS-CoV-2 S protein in place of the endogenous glycoprotein G were performed as previously described (19). Serial dilutions of MAbs were incubated with 10^4^ PFU of VSV-SARS-CoV-2-S_△21_ virus for 1 h at 37°C. Antibody-virus complexes were added to Vero E6 cells and incubated for 7.5 h at 37°C. Cells were then fixed and stained with Hoechst 33342 nuclear stain (Invitrogen). Images were acquired with the InCell 2000 Analyzer (GE Healthcare) automated microscope to visualize nuclei and infected cells (enhanced green fluorescent protein [eGFP]-positive cells). Images were analyzed in InCell Analyzer 1000 Workstation Software (GE Healthcare), and data were processed using Prism version 8 (GraphPad Software, Inc.).

### Virus titration assays.

Plaque assays were performed on Vero E6 cells or Vero-Creanga cells in 24-well plates. Lung tissue homogenates or nasal washes were serially diluted 10-fold, starting at 1:10, in cell infection medium (DMEM plus 2% FBS plus 100 U/ml of penicillin-streptomycin). Two hundred fifty microliters of the diluted virus was added to a single well per dilution per sample. After 1 h at 37°C, the inoculum was aspirated, the cells were washed with PBS, and a 1% methylcellulose overlay in MEM supplemented with 2% FBS was added. Seventy-two hours after virus inoculation, the cells were fixed with 4% formalin, and the monolayer was stained with crystal violet (0.5% [wt/vol] in 25% methanol in water) for 1 h at 20°C. The number of plaques were counted and used to calculate the plaque-forming units per milliliter (PFU/ml).

The 50% tissue culture infectious dose (TCID_50_) was determined in confluent Vero E6 cells in 96-well flat-bottom tissue culture plates. Prior to infection, cells were washed once with PBS and overlaid with infection medium (DMEM supplemented with 4.5 g/liter d-glucose, 100 mg/liter sodium pyruvate, 2% FBS, 100 U/ml penicillin-streptomycin, and 25 mM HEPES). Cells were incubated with serial half-log diluted samples (hamster nasal washes and tissue homogenates) at 37°C for 72 h. Cytopathic effect was monitored to determine the endpoint of infection, and virus titers (log_10_ TCID_50_/ml) were calculated by the Reed-Muench method (32).

To quantify viral load in nasal swabs, lung tissue homogenates, and nasal washes, RNA was extracted from 100-μl samples using E.Z.N.A. Total RNA kit I (Omega) and eluted with 50 μl of water. Four microliters of RNA was used for real-time quantitative RT-PCR (qRT-PCR) to detect and quantify N gene of SARS-CoV-2 using TaqMan RNA-to-CT one-step kit (Thermo Fisher Scientific) as described (33) using the following primers and probes: forward, GACCCCAAAATCAGCGAAAT; reverse, TCTGGTTACTGCCAGTTGAATCTG; probe, ACCCCGCATTACGTTTGGTGGACC; 5′Dye/3′Quencher, 6-carboxyfluorescein (6-FAM)/ZEN/Iowa black fluorescence quencher (IBFQ). Viral RNA was expressed as (N) gene copy numbers per milligram for lung tissue homogenates or per milliliter for nasal swabs and nasal washes, based on a standard included in the assay, which was created via *in vitro* transcription of a synthetic DNA molecule containing the target region of the N gene.

### Histopathology and immunohistochemistry.

Tissues (nasal turbinate and right lung) were fixed in 10% formalin and processed for paraffin embedding. Four-micron sections were stained with hematoxylin and eosin for histopathological examinations. For immunohistochemistry, SARS-CoV-2 N protein was detected using monoclonal antibody (4D11) (34). Images were captured using a Leica DFC 5400 digital camera and were processed using Leica Application Suite v4.13.

### Epitope and escape mutation mapping using yeast-displayed deep mutational scanning libraries.

Antibody epitopes were mapped via a deep mutational scanning approach (21). We previously constructed two independent mutant libraries of the SARS-CoV-2 RBD containing virtually all of the 3,819 possible amino acid mutations in the RBD (20). These libraries were cloned into a vector enabling the expression of RBD on the surface of yeast (35, 36). RBD mutant libraries were previously sorted to enrich for mutant variants that successfully express on the yeast cell surface and bind human ACE2 as described by Greaney et al. (21).

Antibody escape experiments and analysis were performed as described by Greaney et al. (21). Briefly, 9 optical density (OD) units were thawed in 45 ml SD-CAA (6.7 g/liter yeast nitrogen base, 5.0 g/liter Casamino Acids recipe, 1.065 g/liter morpholineethanesulfonic acid [MES], and 2% [wt/vol] dextrose) and grown overnight at 30°C and 275 rpm. Then 33.3 OD units were back diluted into 50 ml SG-CAA plus 0.1% (wt/vol) dextrose (SD-CAA with 2% [wt/vol] galactose and 0.1% [wt/vol] dextrose in place of 2% dextrose) to induce RBD surface expression for 16 to 18 h at 23°C with mild agitation. Twenty-five OD units of cells were washed twice with PBS-BSA (1× PBS with 0.2 mg/ml BSA) and incubated with 400 ng/ml antibody for 1 h at room temperature with gentle agitation, followed by secondary labeling with 1:100 fluorescein isothiocyanate (FITC)-conjugated anti-Myc (CYMC-45F; Immunology Consultants Lab) to quantify RBD expression and 1:200 phycoerythrin (PE)-conjugated goat anti-human IgG (catalog no. 109-115-098; Jackson ImmunoResearch) to measure MAb binding. Flow cytometry and cell sorting were used to select RBD variants (Myc-positive and PE-negative or low [Myc^+^ PE^neg/low^]) that reduce antibody binding via a selection gate drawn to capture wild-type-RBD-expressing cells labeled at 1% of the antibody concentration of the library samples (Fig. S1a and b). Sorts were conducted in duplicate for each antibody using the two independent RBD mutant libraries. For each sample, approximately 10 million RBD-positive (RBD^+^) cells were processed on the flow cytometer, with between 400,000 and 2,800,000 Myc^+^ PE^neg/low^ cells collected per sample (see fractions in Fig. S1b). Antibody-escaped cells were grown overnight in SD-CAA. Plasmids were extracted from the Myc^+^ PE^neg/low^ (antibody-escaped) and preselected yeast populations. Unique identifier barcode sequences that were previously linked to RBD mutant variants (20) were amplified via PCR and sequenced on an Illumina HiSeq 2500.

Read counts were parsed to determine the frequency of RBD variant v in the preselection ($\left(f_{v}^{\text{pre}}\right)$) and antibody-escape ($f_{v}^{\text{post}}$) samples, with a pseudocount of 0.5 added to preselection and antibody-escape read counts. Per-variant escape fraction $\left(E_{v}\right)$, reflecting the fraction of cells with an RBD genotype that fall into the antibody escape bin, was calculated as

$$E_{v}=\frac{F\times f_{v}^{\text{post}}}{f_{v}^{\text{pre}}}$$

where *F* represents the total fraction of the library that escapes binding, as determined experimentally (percentages given in Fig. S1B). To eliminate genotypes that escape antibody due to global loss of folding or function, we eliminated any variants where the per-variant measurement or any of the component amino acid mutations fell below phenotypic thresholds for binding and expression as determined in our prior deep mutational scan (20), eliminating variants and mutations with expression scores of less than –1 and binding scores of less than –2.35 (the binding level of the RaTG13 homolog RBD). Global epistasis models were used to decompose single-mutant escape fractions from per-variant escape scores as described by Greaney et al. (21). Final escape fraction scores were well correlated between replicates at the level of individual mutation effects (Fig. S1c) and the sum of mutation effects per RBD site (Fig. 1e). Final escape scores were taken as the averages of our duplicate library values. Sites of strong escape from each antibody were determined heuristically as sites whose summed mutational escape scores were at least 10 times the median sitewise sum of selection and within 10-fold of the sitewise sum of the most strongly selected site.

### Enzyme-linked immunosorbent assay.

Ninety-six-well microtiter plates (Nunc MaxiSorp; Thermo Fisher Scientific) were coated with 100 μl recombinant SARS-CoV-2 RBD at a concentration of 1 μg/ml in 1× PBS (Gibco) at 4°C overnight; negative-control wells were coated with 1 μg/ml BSA (Sigma). The plates were blocked for 1.5 h at room temperature with 280 μl blocking solution (1× PBS supplemented with 0.05% Tween 20 [Sigma] and 10% FBS [Corning]). The MAbs were diluted to a starting concentration of 10 μg/ml, serially diluted 1:3, and incubated for 1.5 h at room temperature. The plates were washed three times with T-PBS (1× PBS supplemented with 0.05% Tween 20), and 100 μl anti-human IgG horseradish peroxidase (HRP) antibody (goat polyclonal; Jackson ImmunoResearch) diluted 1:2,500 in blocking solution or 100 μl HRP-conjugated anti-hamster IgG2/IgG3 antibody (Southern Biotech catalog no. 1935-05) diluted 1:500 in blocking solution was added to each well and incubated for 1 h at room temperature. The plates were washed three times with T-PBS and three times with 1× PBS, and 100 μl peroxidase substrate (SigmaFast *o*-phenylenediamine dihydrochloride; Sigma) was added to each well. The reaction was stopped after 5 min using 100 μl of 1 M hydrochloric acid, and the plates were read at a wavelength of 490 nm using a microtiter plate reader (BioTek).

To evaluate Fc receptor binding of the chimeric hamster MAb (ham-Ig) and the LALA derivatives, 96-well microtiter plates were coated with 0.02 μg per well of recombinant Fc receptor (human FcγRI [CD64], mouse FcγRI [CD64], mouse FcγRIII [CD16], and mouse FcγRIIIb [CD32b]; BioLegend catalog no. 790006, 773806, 790104 and 783306, respectively) in PBS at 4°C overnight. The plates were blocked for 1.5 h at room temperature with 280 μl blocking solution (1× PBS supplemented with 0.05% Tween 20 [Sigma] and 10% FBS [Corning]). The WT and LALA variant ham-Ig MAbs were diluted to a starting concentration of 10 μg/ml, serially diluted 1:3, and incubated for 1.5 h at room temperature. The plates were washed three times with T-PBS (1× PBS supplemented with 0.05% Tween 20), and 100 μl of HRP-conjugated F(ab′)_2_ anti-hamster IgG antibody (Jackson ImmunoResearch catalog no. 306-036-003) diluted 1:1,000 in blocking solution was added to each well and incubated for 1 h at room temperature. The plates were washed three times with T-PBS and three times with 1× PBS, and 100 μl peroxidase substrate (SigmaFast *o*-phenylenediamine dihydrochloride; Sigma) was added to each well. The reaction was stopped after 5 min using 100 μl of 1 M hydrochloric acid, and the plates were read at a wavelength of 490 nm using a microtiter plate reader (BioTek).

### Quantification and statistical analyses.

Statistical significance was assigned when *P* values were <0.05 using GraphPad Prism version 9.0. Tests, number of animals (*n*), median values, and statistical comparison groups are indicated in the figure legends. Analysis of weight change was determined by one-way or two-way analysis of variance (ANOVA). Changes in infectious virus titer, viral RNA levels, or inflammatory gene expression were compared to isotype-treated animals and were analyzed by one-way ANOVA that is corrected for multiple comparison by controlling the false discovery rate. The Cohen’s *d* value was calculated to measure the effect size of the treatment.

### Data availability.

The complete computational pipeline used to analyze these epitope mapping experiments, and raw and processed data can be found on GitHub at https://github.com/jbloomlab/SARS-CoV-2-RBD_MAP_Ellebedy_Abs. Raw Illumina sequencing data are available from the NCBI SRA at BioSample PRJNA741371 under BioProject PRJNA639956.
