# Supplementary figures and images for: Neutralizing Monoclonal Antibodies That Target the Spike Receptor Binding Domain Confer Fc Receptor-Independent Protection against SARS-CoV-2 Infection in Syrian Hamsters
Source: mBio. 2021 Sep 14;12(5):e02395-21. doi: 10.1128/mBio.02395-21 (PMC8546861; doi:10.1128/mBio.02395-21)

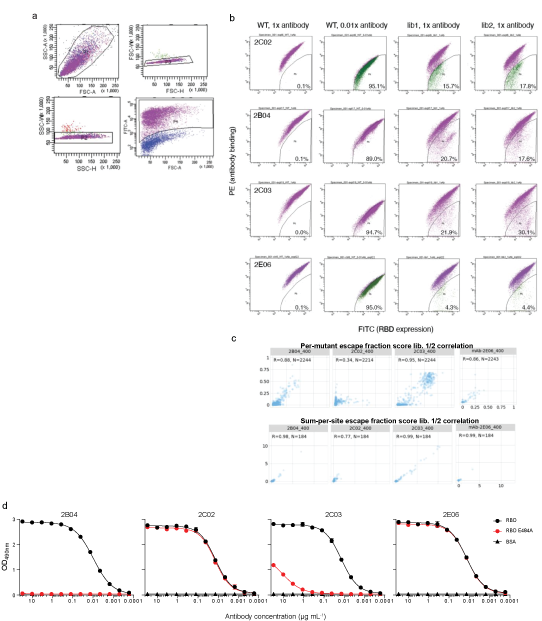

Supplement: FIG S1 [file mbio.02395-21-sf001.tif]

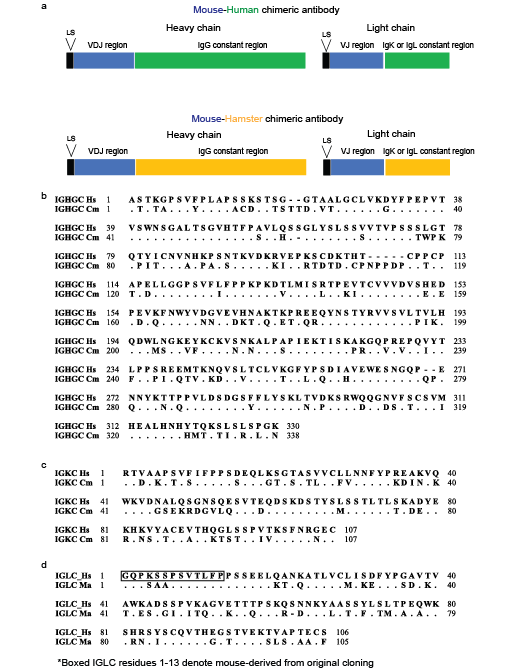

Supplement: FIG S2 [file mbio.02395-21-sf002.tif]
